# Observation of ultrahigh mobility surface states in a topological crystalline insulator by infrared spectroscopy

**DOI:** 10.1038/s41467-017-00446-2

**Published:** 2017-08-28

**Authors:** Ying Wang, Guoyu Luo, Junwei Liu, R. Sankar, Nan-Lin Wang, Fangcheng Chou, Liang Fu, Zhiqiang Li

**Affiliations:** 1 National High Magnetic Field Laboratory, Tallahassee, FL 32310 USA; 2 College of Physical Science and Technology, Sichuan University, Chengdu, Sichuan 610064 China; 3 Department of Physics, Massachusetts Institute of Technology, Cambridge, MA 02139 USA; 4 Department of Physics, Hong Kong University of Science and Technology, Clear Water Bay, Hong Kong, China; 5 Center for Condensed Matter Sciences, National Taiwan University, Taipei, 10617 Taiwan; 6 Institute of Physics, Academia Sinica, Taipei, 11529 Taiwan; 7 International Center for Quantum Materials, School of Physics, Peking University, Beijing, 100871 China

## Abstract

Topological crystalline insulators possess metallic surface states protected by crystalline symmetry, which are a versatile platform for exploring topological phenomena and potential applications. However, progress in this field has been hindered by the challenge to probe optical and transport properties of the surface states owing to the presence of bulk carriers. Here, we report infrared reflectance measurements of a topological crystalline insulator, (001)-oriented Pb_1−*x*_Sn_*x*_Se in zero and high magnetic fields. We demonstrate that the far-infrared conductivity is unexpectedly dominated by the surface states as a result of their unique band structure and the consequent small infrared penetration depth. Moreover, our experiments yield a surface mobility of 40,000 cm^2^ V^−1^ s^−1^, which is one of the highest reported values in topological materials, suggesting the viability of surface-dominated conduction in thin topological crystalline insulator crystals. These findings pave the way for exploring many exotic transport and optical phenomena and applications predicted for topological crystalline insulators.

## Abstract (web summary)

Probing optical and transport properties of the surface states in topological crystalline insulators remains a challenge. Here, Wang et al. demonstrate that the far-infrared conductivity of Pb_1−*x*_Sn_*x*_Se (*x* = 0.23−0.25) single crystals is dominated by the surface states where carriers show a high surface mobility of 40,000 cm^2^ V^−1^ s^−1^.

## Introduction

Recently, a new class of insulators called topological crystalline insulators (TCIs) were predicted and observed in IV–VI semiconductors^1–8^, which have attracted tremendous scientific interest^8–17^. These materials host gapless Dirac-like surface states (SS) that are protected by crystalline symmetry^1, 2^ instead of time-reversal symmetry^18–20^. Consequently, breaking the crystal symmetry can open a band gap in these SS^3, 12^, offering new opportunities for band gap engineering by strain or structural distortion. Moreover, compared with Z_2_ topological insulators^18–20^, the (001) SS of TCIs have been predicted to exhibit a wider range of tunable electronic properties under many types of perturbations breaking the crystalline symmetry, such as interface superconductivity^9^, spin-filtered edge states^10^, quantum anomalous Hall effect^11^, Weyl fermions^13^, and valley-dependent optical properties^14^. Therefore, TCIs are emerging as a very versatile material system not only for exploring topological quantum phenomena, but also for potential device applications in electronics, spintronics, and optoelectronics^8–14^. Several novel characteristics of TCIs have been revealed by surface sensitive probes^3–6, 12^. However, the transport and optical properties of the (001) SS, which are fundamentally important and arguably most relevant to applications^8–14^, have remained especially challenging to measure because of the overwhelming effects of bulk carriers in previous studies^15–17^. This has seriously hampered the progress in this field.

Here, we present infrared (IR) reflectance measurements of a TCI, Pb_1−*x*_Sn_*x*_Se (*x* = 0.23−0.25) single crystals with (001) surface in zero and high magnetic fields. From the band gap, Fermi velocity and Fermi energy of the bulk bands determined from our data, the bulk Drude spectral weight (SW) can be estimated, which is found to be much less than the measured Drude weight, indicating substantial contributions from surface carriers. Secondly, the spectral features in magneto-reflectance spectra below 25 meV can be attributed to a dominant resonance at $\omega _{\rm{c}}^{{\rm{ss}}} \propto B$ based on theoretical study of cyclotron resonance (CR) of the SS, the frequency of which obtained from our data ($\omega _{\rm{c}}^{{\rm{ss}}}$) is quantitatively consistent with those estimated from previous scanning tunneling microscopy (STM) and angle-resolved photoemission spectroscopy (ARPES) experiments. Moreover, the SW of the dominant resonance in magnetic field is in accord with the extra Drude weight in zero field besides the bulk contribution. Above all, we demonstrate that the resonance at $\omega _{\rm{c}}^{{\rm{ss}}}$ in field is well below the energy range of all Landau level (LL) transitions (including CR) from the bulk states, so it can only be assigned to the SS. Therefore, all these findings taken together provide robust evidence for SS in Pb_1−*x*_Sn_*x*_Se. Remarkably, we find that the IR conductivity of Pb_1−*x*_Sn_*x*_Se is dominated by the SS in the far-IR range (7–25 meV or 2–6 THz) despite the presence of bulk carriers. We show that this unexpected property arises from the unique band structure of the SS and the resultant high surface carrier density and small IR penetration depth. Furthermore, our experiments yield a surface mobility of ~40,000 cm^2^ V^−1^ s^−1^ based on analysis of CR mode of the SS, which is 1–2 orders of magnitude higher than that in TCI thin films^21–23^ and among the highest reported values in topological materials. The ultrahigh surface mobility and other transport parameters obtained here suggest that surface-dominated transport can be achieved in (001)-oriented Pb_1−*x*_Sn_*x*_Se crystals with sub-micron thickness. Our findings open up opportunities for exploring many exotic transport and optical phenomena and applications predicted for TCIs^8–14^, ranging from quantum anomalous Hall effect and Weyl fermions to spintronics and valleytronics.

## Results

### IR spectra of Pb_1−*x*_Sn_*x*_Se

**Fig. 1:**
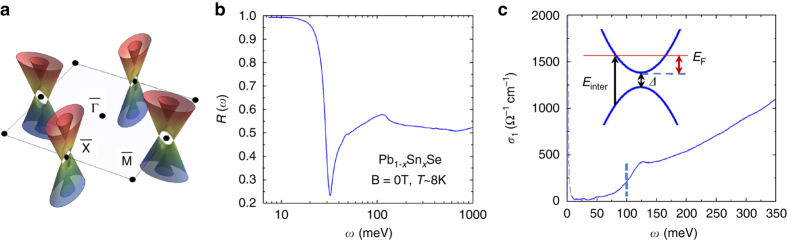
IR spectra of Pb_1−*x*_Sn_*x*_Se (*x* = 0.23–0.25) in zero magnetic field. **a** Schematic band structure of the SS and the surface Brillouin zone. **b** IR reflectance spectrum *R*(*ω*) at *T* = 8 K. **c** The real part of the optical conductivity *σ*_1_(*ω*) at *T* = 8 K. The *vertical dashed line* around 100 meV indicates *E*_inter_. The *inset* shows a schematic of the band structure of the bulk states and the onset of inter-band transitions at *E*_inter_

In this work, Pb_1−*x*_Sn_*x*_Se single crystals with an actual composition of *x* = 0.23−0.25 were investigated, which are in the TCI phase and host gapless SS^4^ (Fig. 1a). The actual composition is determined from the bulk band gap^4^ as discussed below. These materials have a direct bulk band gap located at four L points (valleys) in the three-dimensional (3D) Brillouin zone. Our samples are n-doped with the Fermi energy *E*_F_ in the bulk conduction band^3^. The IR reflectance spectra *R*(*ω*) of Pb_1−*x*_Sn_*x*_Se crystals with (001) surface were measured in zero field and in magnetic field applied perpendicular to the surface of the samples. Figure 1 depicts the *R*(*ω*) spectrum and the dissipative part of the optical conductivity *σ*_1_(*ω*) in zero field at T = 8 K (see “Methods”). The *R*(*ω*) spectrum shows a typical metallic behavior: *R*(*ω*) is very close to 1 below 15 meV; at higher energy the reflectance is gradually depressed toward a plasma minimum at about 30 meV, followed by a peak around 120 meV. The energy of the plasma minimum observed here is much lower than those in earlier IR studies^16, 17^, indicating much lower total carrier density in our samples due to low density of Se vacancy defects (see “Methods” and Supplementary Note 1). The zero field *σ*_1_(*ω*) spectrum exhibits a Drude component below 25 meV and a threshold-like feature around 100 meV. The narrow plasma minimum in *R*(*ω*) with a half width ~3 meV is a direct manifestation of the very narrow Drude peak in *σ*_1_(*ω*), which is corresponding to a very low scattering rate. The threshold feature in *σ*_1_(*ω*) can be assigned to the onset of inter-band transitions for the bulk around ${E_{{\rm{inter}}}} \approx {\it{\Delta}} + {E_{\rm{F}}}\left( {1 + \frac{{{m_{\rm{c}}}}}{{{m_{\rm{v}}}}}} \right)$ as illustrated in the *inset* of Fig. 1c, where *Δ* is the bulk band gap, the Fermi energy *E*_F_ is defined with respect to the bottom of the conduction band, *m*_c_ and *m*_v_ are effective masses of the conduction and valence band, respectively. The experimental absorption coefficient spectrum also exhibits a similar threshold feature in the same energy range. It is shown that in doped semiconductors the absorption coefficient can be written as^24, 25^ $\alpha \left( \omega \right) \propto {\alpha _0}\left( \omega \right)\left[ {1 - f(\omega ,{E_{\rm{F}}},T)} \right]$, where ${\alpha _0}\left( \omega \right) \propto \sqrt {\hbar \omega - {\it{\Delta}} /\hbar \omega }$ is the absorption coefficient for the undoped material, $f\left( {\omega ,{E_{\rm{F}}},T} \right) = \left[ {1 + \exp \left( {\frac{{\hbar \omega - {E_{{\rm{inter}}}}}}{{\left( {1 + \frac{{{m_{\rm{c}}}}}{{{m_{\rm{v}}}}}} \right){k_{\rm{B}}}T}}} \right)} \right]^{ - 1}$ is the Fermi distribution, $\hbar$ is Planck’s constant divided by 2π, *k*_B_ is the Boltzmann constant. Fitting the experimental absorption coefficient using the equation above, we find *E*_inter_~100 meV as indicated by the *dashed line* in Fig. 1c.

**Fig. 2:**
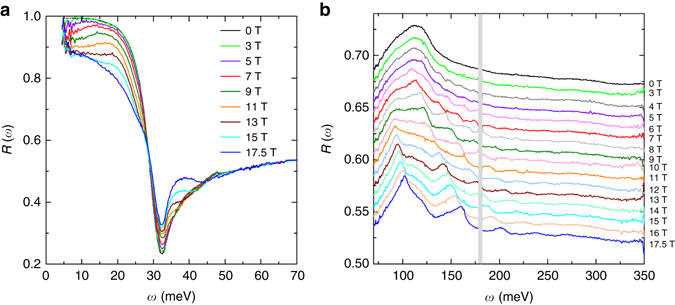
IR reflectance spectra in magnetic fields. **a**, **b** Reflectance spectra *R*(*ω*, *B*) in several magnetic fields at T~4.5 K. For clarity the spectra in **b** are displaced from one another by 0.01 with the spectrum at *B* = 17.5 T shown at its actual value. The *gray* area around 175 meV is the energy range in which no data can be obtained due to the absorption of the optical window in our setup

**Fig. 3:**
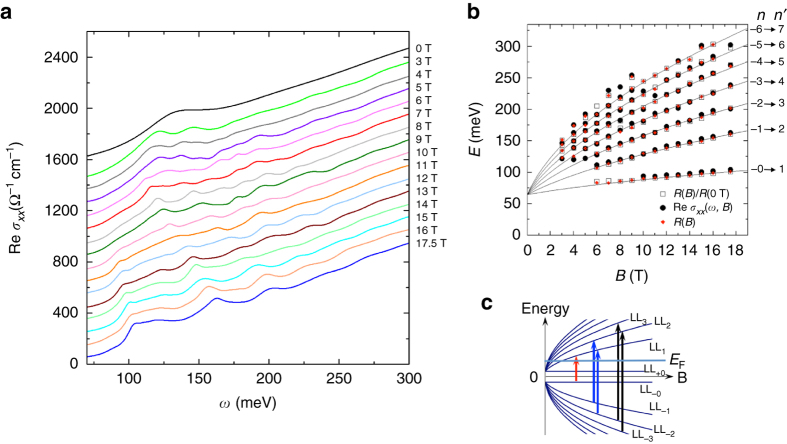
LL transitions of bulk states. **a** The real part of the optical conductivity Re *σ*_*xx*_(*ω*) in several magnetic fields. For clarity the spectra are displaced from one another by 100 with the spectrum at *B* = 17.5 T shown at its actual value. **b** The energies of the observed transitions as a function of magnetic field. *Symbols*: data. *Solid lines*: fits to the data using Eq. (1) discussed in the text with *Δ* = 64.5 meV and *v*_F_ = 0.4 × 10^6^ m s^−1^. The observed resonances can be assigned to LL transitions T_*n*_, which is due to transitions LL_-(*n*−1)_→LL_*n*_ and LL_-*n*_→LL_*n*−1_ for *n *> 1 and LL_−0_→LL_1_ for *n* = 1. **c** Schematic of LLs of the bulk states. The *arrows* illustrate T_1_–T_3_ shown in **b**

More insights into the band structure of Pb_1−*x*_Sn_*x*_Se can be provided by a systematic investigation of the magneto-reflectance *R*(*ω*, *B*) spectra displayed in Fig. 2 (see “Methods”). Strikingly, the zero field *R*(*ω*) spectrum is strongly modified by a magnetic field. The *R*(*ω*, *B*) spectra are strongly suppressed below 25 meV in magnetic fields and no longer extrapolate to unity in the limit of *ω*→0. Moreover, a series of new resonance features are observed in *R*(*ω*, *B*) extending up to 300 meV, all of which evolve systematically with magnetic fields. In order to acquire a complete understanding of the features in *R*(*ω*, *B*) spectra, the optical conductivity in magnetic field was extracted from an analysis of *R*(*ω*, *B*) using the magneto-Drude-Lorentz model^26–28^ (see “Methods”). Figure 3a depicts the real part of the optical conductivity Re *σ*_*xx*_(*ω*, *B*) from 50 to 300 meV, which exhibit several resonance (absorption) peaks systematically shifting to higher energies with increasing magnetic field. The energies (*E*) of the absorption features in Re *σ*_*xx*_(*ω*, *B*) are displayed in Fig. 3b, which show good agreement with the energies of the resonance features in *R*(*ω*, *B*). The field-dependent absorption features can be assigned to LL transitions. Interestingly, the observed transitions are not equally spaced in energy in any spectrum, which is in stark contrast to the behavior of systems with quadratic energy-momentum dispersion. We find that the observed resonance features in Re *σ*_*xx*_(*ω*, *B*) can be well described by LL transitions of 3D massive Dirac fermions^8, 15^, whose LL energies have the form:

1 $${E_n}\left( {{k_z}} \right) = \pm {\delta _{n,0}}\sqrt {{{\left( {{\it{\Delta}} /2} \right)}^2} + {{\left( {\hbar {v_{\rm{F}}}{k_z}} \right)}^2}} \\ + \,{\rm{sgn}}\left( n \right)\sqrt {2e\hbar v_{\rm{F}}^2B\left| n \right| + {{\left( {{\it{\Delta}} /2} \right)}^2} + {{\left( {\hbar {v_{\rm{F}}}{k_z}} \right)}^2}} $$

which have two zeroth LLs labeled as *n* = +0 and *n* = −0. Here, the integer *n* is LL index, *k*_*z*_ is momentum along the direction of the magnetic field, *e* is the elementary charge, *v*_F_ is the Fermi velocity, *δ* is the Kronecker delta function, and sgn(*n*) is the sign function. Theoretical studies showed that the optical conductivity has sharp peaks at energies of LL transitions at *k*_z_ = 0 because of singularities in the joint-density-of-states between LLs at these energies^29^. From the selection rule^29, 30^ for allowed optical transitions from LL_n_ to LL_n′_, Δ*n *= $\left| n \right|$–$\left| {n'} \right|$ = ±1, we find that all the observed resonances can be assigned to allowed LL transitions based on Eq. (1) with *k*_z_ = 0, *Δ*≈64 ± 3 meV, and *v*_F_≈(0.400 ± 0.005) × 10^6^ m s^−1^ (Fig. 3b, c), which are determined from least squares fit of the observed transition energies. This analysis shows that the sharp resonances in Fig. 3a mainly arise from massive Dirac fermions of the bulk states^8, 15^. Since the Sn substitution level directly leads to changes in the bulk band gap, the actual composition of our samples can be determined from *Δ* to be *x* = 0.23−0.25^4^.

### IR signatures of SS

Our data allow us to identify the signatures of SS in Pb_1−*x*_Sn_*x*_Se because all spectroscopic features of the bulk states can be determined. The first evidence for the SS is from an analysis of the Drude SW (area under *σ*_1_(*ω*)) in zero field. The low-energy bulk states can be described by massive Dirac fermions: $E(k) = \pm \sqrt {{\hbar ^2}v_{\rm{F}}^2{k^2} + {{\left( {{\it{\Delta }}/2} \right)}^2}}$, with *Δ* and *v*_F_ given above. We can estimate *E*_F_ of the bulk states from *E*_inter_≈*Δ* + 2*E*_F_ (note that *m*_c_ = *m*_v_ for massive Dirac fermions) shown in Fig. 1c. Alternatively, the transition LL_−0_→LL_+1_ disappears at *B* < 6 T (Figs. 2, 3), so the Fermi energy can be estimated as *E*_F_~LL_+1_(*B* = 6 T)−*Δ*/2 using Eq. (1). Either method yields *E*_F_≈17 ± 2 meV. From the band dispersion and *E*_F_, we obtain *k*_F_≈0.014 ± 0.001 Å^−1^. Recent transport studies found that the bulk Fermi surface pockets of Pb_1−*x*_Sn_*x*_Se in the TCI phase are nominally spherical^15^, so the bulk carrier density can be estimated by ${n_{{\rm{bulk}}}} = \frac{1}{{{{\left( {2\pi } \right)}^3}}}\frac{4}{3}\pi k_{\rm{F}}^3{g_{\rm{s}}}{g_{\rm{v}}} \approx \left( {3.7 \pm 1.1} \right) \times {10^{17}}{\rm{c}}{{\rm{m}}^{{\rm{ - 3}}}}$, where *g*_s_ = 2 and *g*_v_ = 4 are the bulk spin and valley degeneracy, respectively. Similarly low carrier density has been reported by previous transport experiments^15^. Moreover, the effective mass of massive Dirac fermions at *E*_F_ can be calculated from ${m_{{\rm{bulk}}}} = \frac{{{E_{\rm{F}}} + {\it{\Delta }}/2}}{{v_{\rm{F}}^2}} \approx 0.054 \pm 0.003{m_e}$, where *m*_e_ is bare electron mass. The SW of the Drude absorption is related to the bare plasma frequency $\omega _{\rm{P}}^2 = 4\pi {e^2}n/m$ by^31^:

2 $${\rm{SW}} = \mathop {\int}\limits_0^{{\omega _0}} {{\sigma _1}\left( \omega \right){\rm{d}}\omega = \frac{{\pi {\Omega ^{ - 1}}}}{{120}}} \,\,\omega _{\rm{P}}^2$$

where *ω*_0_ is a cut-off frequency separating the Drude component from the inter-band transitions, Ω is Ohm, *σ*_1_ is in Ω^−1^ cm^−1^, and all frequencies are in cm^−1^. From *n*_bulk_, *m*_bulk_, and Eq. (2), we estimate that ${\rm{S}}{{\rm{W}}_{{\rm{bulk}}}} \approx \left( {1.6 \pm 0.5} \right) \times {10^4}{\Omega ^{ - 1}}{\rm{c}}{{\rm{m}}^{ - 2}}$ for the bulk states from calculating *ω*_P, bulk_. On the other hand, the total Drude SW can be determined by the total plasma frequency *ω*_P_ of the Drude component in the experimental data using ${\rm{S}}{{\rm{W}}_{{\rm{total}}}} = \frac{{\pi {\Omega ^{ - 1}}}}{{120}}\omega _{\rm{P}}^2$, where *ω*_P_ is related to the screened plasma frequency ${\tilde \omega _{\rm{P}}}$ by ${\omega _{\rm{P}}} = {\tilde \omega _{\rm{P}}}\sqrt {{\varepsilon _\infty }}$, with ${\tilde \omega _{\rm{P}}}\&sim;$260 cm^−1^ (32.4 meV) corresponding to the plasma minimum in *R*(*ω*) and *ε*_∞_ representing all electronic contributions to the dielectric constant other than the Drude component. Therefore, the total plasma frequency *ω*_P_ can be obtained from *ε*_∞_, which is determined by all Lorentzian oscillators obtained from Drude-Lorentz fit of the entire R(*ω*) spectrum (see Supplementary Note 5). We find that *ε*_∞_~45 ± 9, so SW_total_≈(7.9 ± 1.6) × 10^4^ Ω^−1^ cm^−2^. A comparison between SW_bulk_ and SW_total_ shows that the bulk contribution can only account for about 20% of the total Drude SW of the system. We emphasize that the total Drude SW and that from bulk states are both accurately determined from our data, which strongly suggests dominant surface contribution to the total Drude absorption in zero field.

**Fig. 4:**
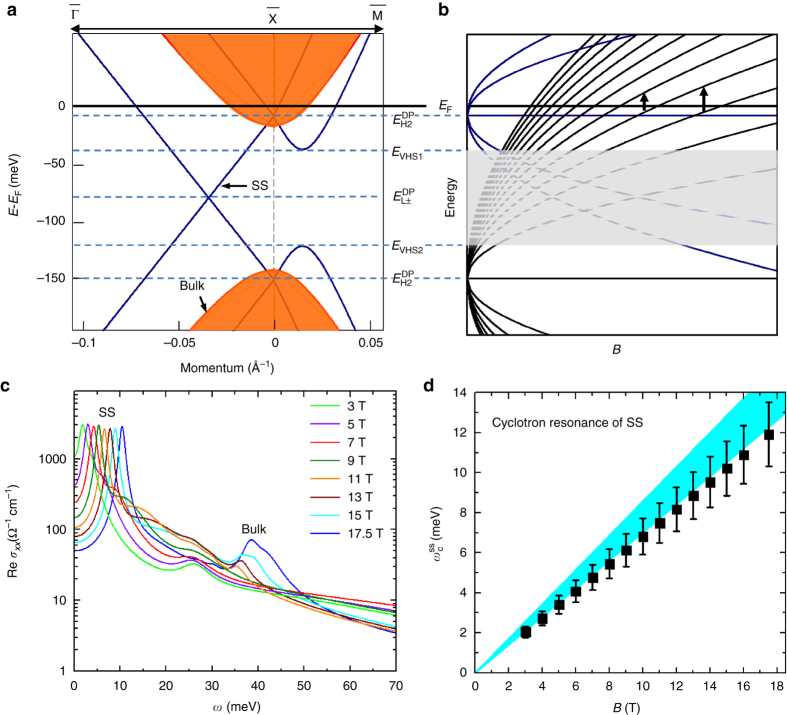
IR conductivity of SS in magnetic fields. **a** A schematic of the SS band structure (*dark blue*) and that of the bulk (*red*) along two high-symmetry directions for one of the four Dirac cones inside the surface Brillouin zone. $E_{{\rm{H1}}}^{{\rm{DP}}}$, $E_{{\rm{H2}}}^{{\rm{DP}}}$, and $E_{{\rm{L}} \pm }^{{\rm{DP}}}$ are energies of the Dirac points associated with the two Dirac cones located at the $\overline {\rm{X}}$ points and the Dirac nodes along the $\overline {\Gamma {\rm{X}}}$ line, respectively. *E*_VHS1_ and *E*_VHS2_ are energies of the two Van Hove singularities in the band structure. **b** A schematic of LLs of the SS in the same energy scale as that in **a**. While the LLs between *E*_VHS1_ and *E*_VHS2_ in the *gray-shaded area* have nontrivial dispersions^32^ (not shown here), the LLs near *E*_F_ are well approximated by LLs of two independent Dirac cones at $E_{{\rm{H}}1}^{{\rm{DP}}}$ and $E_{{\rm{H}}2}^{{\rm{DP}}}$ as illustrated by the *dashed lines* in the *shaded* area. The CR of the SS associated with the Dirac cone at $E_{{\rm{H}}2}^{{\rm{DP}}}$ is shown by the *vertical arrows*. **c** The real part of the model optical conductivity Re *σ*_xx_(*ω*) in several magnetic fields that are used to simulate the *R*(*ω*, *B*) spectra below 70 meV. The strong peak below 15 meV in Re *σ*_*xx*_(*ω*) arises from CR of the SS, which represents the spatially averaged 3D optical conductivity of the SS within the IR penetration depth. **d** The CR energy of SS $\omega _{\rm{c}}^{{\rm{ss}}}$ as a function of magnetic field. The *error bars* represent the range of $\omega _{\rm{c}}^{{\rm{ss}}}$ values that can be used in our model to produce excellent fit of the *R*(*ω*, *B*) data within experimental uncertainties as discussed in Supplementary Note 2. The *shaded area* indicates the range of $\omega _{\rm{c}}^{{\rm{ss}}}$ values estimated from previous STM and ARPES experiments

The low-energy magneto-reflectance of Pb_1−*x*_Sn_*x*_Se provides further evidence for the SS. The systematic suppression of *R*(*ω*, *B*) spectrum with *B* field below 25 meV suggests that the system becomes more insulating with increasing field. This is reminiscent of the typical behavior of CR. For instance, similar low-energy behaviors in *R*(*ω*, *B*) were also observed in graphite^27^ due to CR, which manifests itself in Re *σ*_*xx*_(*ω*) as a peak at energies of *E*∝*B*. We now show that the spectral feature below 25 meV in *R*(*ω*, *B*) and its evolution with *B* field are unambiguous signatures of CR from the SS (Supplementary Notes 2 and 3). As shown in Figs. 1a and 4a, the band structure of SS on the (001) surface features two generations of Dirac fermions^7^, starting from a pair of Dirac cones located at the $\overline X$ points of the surface Brillouin zone with their Dirac points at $E_{{\rm{H}}1}^{{\rm{DP}}}$ and $E_{{\rm{H}}2}^{{\rm{DP}}}$. The hybridization between these two Dirac cones leads to a gap in all directions except along the $\overline {\Gamma {\rm{X}}}$ line (Fig. 1a), where a pair of Dirac points exist that are protected by the (110) mirror symmetry^2, 7^. Recent experiments and theoretical calculations^3, 32, 33^ suggest that $E_{{\rm{H}}1}^{{\rm{DP}}}$ is close to *E*_F_ of the bulk states for Pb_1−*x*_Sn_*x*_Se. In a magnetic field perpendicular to the surface, it is shown^32^ that the LLs of the SS near *E*_F_ are well approximated by LLs of two independent Dirac cones at $E_{{\rm{H}}1}^{{\rm{DP}}}$ and $E_{{\rm{H}}2}^{{\rm{DP}}}$ (Fig. 4b), which have energies of ${E_n} = {\rm{sgn}}\left( n \right)\sqrt {2e\hbar {{\left( {\bar v_{\rm{F}}^{{\rm{SS}}}} \right)}^2}B\left| n \right|}$ with respect to each Dirac point and $\bar v_{\rm{F}}^{{\rm{SS}}}\sim 0.4 \times {10^6}$m s^−1^ ^3, 32, 33^. Within this picture, a CR mode due to the intra-band LL transition associated with the Dirac cone at $E_{{\rm{H}}2}^{{\rm{DP}}}$ is expected at low energy (Fig. 4b). The surface Fermi energy with respect to $E_{{\rm{H}}2}^{{\rm{DP}}}$ is estimated to be $E_{\rm{F}}^{{\rm{SS}}}\sim136 \pm 14\,{\rm{meV}}$ based on STM and ARPES experiments^3, 32, 33^, from which the energy of the surface CR mode is expected to be $\omega _{\rm{c}}^{{\rm{ss}}} = eB/{m_{ss}}$ with ${m_{{\rm{ss}}}} = E_{\rm{F}}^{{\rm{SS}}}/{\left( {\bar v_{{F}}^{{\rm{SS}}}} \right)^2}$ = 0.15 ± 0.015 *m*_e_. LL transitions of SS associated with the Dirac cone at $E_{{\rm{H}}1}^{{\rm{DP}}}$ can also contribute to the *R*(*ω*, *B*) spectra at $\sqrt {2e\hbar {{\left( {\bar v_F^{{\rm{SS}}}} \right)}^2}B}$ or higher energy, but it is very difficult to separate them from bulk LL transitions that are in the same energy range. It is shown^32^ that LLs associated with the Dirac cone along the $\overline {\Gamma {\rm{X}}}$ line are restricted in the energy range between *E*_VHS1_ and *E*_VHS2_ in Fig. 4a, b, so these LLs below *E*_F_ cannot be probed from optical measurements, which requires optical transitions from a LL below *E*_F_ to one above *E*_F_. To examine the IR signatures of the surface CR mode, the magneto-Drude-Lorentz model is used to simulate the *R*(*ω*, *B*) spectra (see “Methods”). The real and imaginary parts of *σ*_*xx*_(*ω*), *σ*_*xy*_(*ω*), *σ*_*+*_(*ω*), and *σ*_*−*_(*ω*) in representative models are displayed and discussed in Supplementary Note 2. We find that the principle features of *R*(*ω*, *B*) can be reproduced by model optical conductivity spectra that are based on the expected behaviors of the surface CR mode^32^: remarkably, a strong resonance peak at $\omega _{\rm{c}}^{{\rm{ss}}}$ in Re *σ*_*xx*_(*ω*, *B*) that changes linearly with *B* field (Fig. 4c) can capture the spectral feature in *R*(*ω*, *B*) below 25 meV and its evolution with *B* field. In particular, the analysis on our data yields *m*_ss_ values in the range of 0.15–0.19 *m*_e_ for the CR mode, which are consistent with those estimated from STM and ARPES experiments^3, 33^ within 15% as shown in Fig. 4d (see Supplementary Note 2). The small deviation between our results and STM and ARPES measurements may arise from the difference in Fermi energy in different samples. Based on LL energies calculated from *Δ* and *v*_F_ for the bulk and Eq. (1), the peak between 30 and 50 meV in Re *σ*_*xx*_(*ω*) spectra can be assigned to the LL_+0_→LL_+1_ transition (intra-band LL transition or CR) from the bulk states for *B *> 6 T, which is responsible for the feature around 37 meV in *R*(*ω*, *B*). Because *E*_F_ is between LL_+0_ and LL_+1_ above *B*~6 T, the bulk CR LL_+0_→LL_+1_ around 37 meV has the lowest energy among all allowed LL transitions for the bulk for *B* > 6 T (Fig. 3c). Therefore, the spectral feature in *R*(*ω*, *B*) below 25 meV can only be assigned to the SS, because it is well below the energy range of all LL transitions (including CR) from the bulk states for *B *> 6 T. Moreover, from the area under the resonance at $\omega _{\rm{c}}^{{\rm{ss}}}$ in Re *σ*_*xx*_(*ω*), the SW of this resonance is found to be SW_SS_~(4.4 ± 0.9) × 10^4^ Ω^−1^ cm^−2^, which is in agreement with the extra Drude SW in zero field besides the bulk contribution ~(6.3 ± 2.1) × 10^4^ Ω^−1^ cm^−2^. This agreement provides further support for our identification of the surface CR mode. Therefore, our data in zero and high magnetic fields taken together provide robust evidence for the IR signatures of SS in Pb_1−*x*_Sn_*x*_Se.

### SS dominated far-IR conductivity

Strikingly, the far-IR conductivity of (001)-oriented Pb_1−*x*_Sn_*x*_Se is dominated by the SS in the energy range of the Drude absorption in zero field and that of the surface CR mode in magnetic field, which is around 7–25 meV or 2–6 THz. We now show that this is a consequence of the unique band structure of the (001) SS. The SS of our n-doped samples has two Fermi surfaces associated with the two Dirac cones at $E_{{\rm{H}}1}^{{\rm{DP}}}$ and $E_{{\rm{H}}2}^{{\rm{DP}}}$ (Fig. 4a). In particular, the latter Dirac cone gives rise to a large Fermi surface with ${\overline{k}}_{\rm{F}}^{{\rm{SS}}}\sim {E}_{\rm{F}}^{{\rm{SS}}}/\left( {\hbar \overline v _{\rm{F}}^{{\rm{SS}}}} \right)\sim 0.052 \pm 0.005\,{{\AA}^{ - 1}}$ ^3, 32, 33^. Neglecting the small contribution from the Fermi surface associated with the Dirac cones at $E_{{\rm{H}}1}^{{\rm{DP}}}$ and taking into account *g*_s_ = 1 and *g*_v_ = 2 for the SS, we estimate that the large SS Fermi surface leads to a very high surface carrier density $n_{{\rm{SS}}}^{2{\rm{D}}}\sim\left( {4.2 \pm 0.8} \right) \times {10^{12}}\,{\rm{c}}{{\rm{m}}^{ - 2}}$, which is more than one order of magnitude higher than that of the (111) surface in TCIs^21, 34^. The large value of $n_{{\rm{SS}}}^{2{\rm{D}}}$ of the (001) surface is critical for achieving surface dominated far-IR conductivity in Pb_1−*x*_Sn_*x*_Se despite the presence of bulk carriers. Our measurements only probe the region of the sample within the IR penetration depth *δ*. An estimate for the length scale of *δ* for our samples between 7 and 25 meV can be obtained from a SW analysis. The Drude weight of the (001) SS can be estimated as ${e^2}E_{\rm{F}}^{{\rm{SS}}}{g_{\rm{s}}}{g_{\rm{v}}}/\left( {8{\hbar ^2}} \right)$ ^35^, which only includes contributions from the massless Dirac fermions associated with the Dirac cone at $E_{{\rm{H2}}}^{{\rm{DP}}}$. The same surface Drude weight can be obtained from SW_SS_ *δ* based on our IR measurements, so the averaged length scale of *δ* between 7 and 25 meV is estimated to be 13 ± 4 nm for our samples. It can be shown that *δ* is comparable to the penetration depth of the SS (see “Methods”). This suggests that the high-carrier density of the (001) SS leads to strong IR absorption and attenuates the IR light within a very small penetration depth, which effectively reduces the bulk contribution to the far-IR conductivity. It is instructive to compare our results with a recent IR study of (111)-oriented TCI thin films^34^, which reported substantially lower surface SW compared to the bulk contribution. It is found that the (111) surface carrier density is ten times lower and the IR penetration depth is much larger than those in our samples^34^, owing to a much smaller *k*_F_ for the (111) SS. These results are consistent with our findings that the high surface carrier density reduces the IR penetration depth and thus the bulk contribution to the overall far-IR conductivity in (001)-oriented Pb_1−*x*_Sn_*x*_Se. Therefore, the unique band structure of (001) SS plays a crucial role in achieving surface-dominated far-IR conductivity observed in our study. The large difference in the IR properties of (001) and (111) surfaces is a new aspect in TCIs compared to those in Z_2_ topological insulators^36–39^.

## Discussion

Our study sheds new light on the mobility of the (001) SS of Pb_1−*x*_Sn_*x*_Se. From half width of the surface CR mode in Re *σ*_*xx*_(*ω*), the scattering rate of the SS is estimated to be 1/*τ*_SS_~1.2 ± 0.6 meV, which clearly manifest itself as the very narrow dip feature around 32 meV in *R*(*ω*, *B*) (Supplementary Note 4). Therefore, the mobility of the SS can be estimated: ${\mu _{{\rm{SS}}}} = e\bar v_{\rm{F}}^{{\rm{SS}}}{\tau _{{\rm{SS}}}}/\left( {\hbar \bar k_{\rm{F}}^{{\rm{SS}}}} \right) = e{\tau _{{\rm{SS}}}}/{m_{{\rm{ss}}}}\sim40,000\,{\rm{c}}{{\rm{m}}^2}{V^{ - 1}}{s^{ - 1}}$ with an uncertainty of about 50%. Such a high surface mobility is very promising for studying the intrinsic physics of TCIs. Detailed analysis on the scattering rate and mobility from previous IR study of LL transitions in graphene^40^ support our estimation of surface mobility in TCIs since both systems feature massless Dirac fermions (Supplementary Note 4). In contrast, previous studies on TCI thin films reported surface mobility values in the range of 100–2000 cm^2^ V^−1^ s^−1^ ^21–23^ and a broad surface IR absorption feature that is ten times wider than that in our data^34^. The low mobility values of these TCI thin films are very likely limited by sample quality. We note that the small scattering rate and high mobility of the (001) SS also play an important role in leading to strong surface absorption in a narrow energy range in far-IR and thus a small IR penetration depth, which is responsible for the observed surface-dominated IR conductivity.

A long-standing challenge in the research of TCIs as well as Z_2_ topological insulators is to realize surface-dominated transport^41–45^. Our study elucidates the surface and bulk contributions to electronic transport. From the effective mass of bulk states *m*_bulk_ obtained from zero-field data and the scattering rate estimated from bulk LL transitions 1/*τ*_bulk_~10 meV, the bulk mobility of our samples can be estimated: *μ*_bulk_~13,000 cm^2^ V^−1^ s^−1^. Similarly high mobility has been reported by previous transport experiments^15^. For a sample with thickness *d*, the fraction of surface contribution to the total conductance can be estimated as $n_{{\rm{SS}}}^{2{\rm{D}}}{\mu _{{\rm{SS}}}}e/\left( {n_{{\rm{SS}}}^{2{\rm{D}}}{\mu _{{\rm{SS}}}}e + {n_{{\rm{bulk}}}}d{\mu _{{\rm{bulk}}}}e} \right)$. The mobility and carrier density values obtained in our study suggest that the SS in our samples will contribute to more than 50% of the total conductance in crystals with *d* < 0.4 μm. Surface-dominated transport can be achieved in even thicker crystals if the bulk carrier density is reduced further in future.

In conclusion, our study has revealed several IR signatures of SS in Pb_1−*x*_Sn_*x*_Se, including their contribution to the Drude absorption in zero field and their CR mode in magnetic field, whose SWs are further found to be consistent. Moreover, the frequency of the surface CR mode $\omega _{\rm{c}}^{{\rm{ss}}}$ inferred from our data is in accord with those estimated from theoretical studies and STM and ARPES measurements. Finally, we show that the resonance at $\omega _{\rm{c}}^{{\rm{ss}}}$ in field can only be assigned to the SS, because it is well below the energy range of all bulk LL transitions. Our study demonstrates the viability of surface-dominated IR conductivity and transport, as well as an ultrahigh surface mobility of ~40,000 cm^2^ V^−1^ s^−1^ in Pb_1−*x*_Sn_*x*_Se with (001) surface. We expect that these results can be found in a broad class of IV–VI semiconductor TCIs Pb_1−*x*_Sn_*x*_Se(Te) because of the similar properties of their SS^1–8^. These findings establish Pb_1−*x*_Sn_*x*_Se(Te) thin crystals (potentially with electrostatic gating) as a fertile system for accessing the optical and transport properties of the (001) SS, which is a crucial step for exploring many novel topological phenomena in TCIs and their potential electronic, spintronic, and valleytronic applications^8–14^.

## Methods

### IR measurements and data analysis

Pb_1−*x*_Sn_*x*_Se (*x* = 0.23–0.25) single crystals with centimeter size were grown by the Bridgman growth method. As Pb_1−*x*_Sn_*x*_Se crystals have cubic symmetry, the samples in our study have been chosen from naturally cleaved cubic crystals with easy low indexing (00L) cleavage planes. The carrier density in the crystals is batch (growth)-dependent even for the same nominal Pb/Sn ratio because of the self-doping effect of Se vacancy defects (see Supplementary Note 1). The crystals used in our IR study are from a batch with low-carrier (defect) density. The samples were cleaved in ambient conditions before the first IR measurement, and their exposure time in air was minimized between subsequent measurements. The zero-field reflectance spectrum *R*(*ω*) was measured in the energy range of 7.5–900 meV at a temperature of T = 8 K. The magneto-reflectance ratio spectra *R*(*ω*, *B*)/*R*(*ω*, *B* = 0 T) were measured at T~4.5 K in a superconducting magnet in the Faraday geometry (magnetic field perpendicular to the sample surface). The *R*(*ω*, *B*) spectra were obtained by multiplying the magneto-reflectance ratio spectra by *R*(*ω*) at zero field (Supplementary Note 1). We estimate the experimental uncertainty of the absolute values of *R*(*ω*, *B*) is about 1–1.5%, but the relative change (evolution) of *R*(*ω*, *B*) with magnetic field has a smaller uncertainty owing to the better accuracy of the magneto-reflectance ratio data.

As an approximation for exploring spatially averaged properties of the SS, our data analysis employs a model with uniform carrier density *n*_SS_ for the SS without a sharp surface/bulk interface, which will be elaborated below. In such a model, the IR data can be analyzed using formulas for bulk materials and the resulting optical conductivity contains contributions from both the SS and bulk, with all quantities of the SS being spatially averaged ones along the direction perpendicular to the surface within the IR penetration depth. The complex optical conductivity *σ*(*ω*) = *σ*_1_(*ω*) + *iσ*_2_(*ω*) in zero field at 8 K was obtained from analysis of *R*(*ω*) using Drude-Lorentz model combined with Kramers-Kronig (KK) constrained variational dielectric functions^28^. Alternatively, *σ*(*ω*) in zero field can be obtained from KK transformation of *R*(*ω*) data, in which Hagen–Rubens formula was employed in the low-energy region and the *R*(*ω*) data at 8 K was extend to 5.765 eV using room temperature reflectance data. The two approaches above yield consistent *σ*(*ω*) spectra with identical spectral features. In magnetic field, the R(*ω*, *B*) spectra were analyzed using magneto-Drude-Lorentz model^26^, in which the optical conductivity for right and left circularly polarized light (denoted + and −, respectively) is given by:

3 $${\sigma _ \pm }\left( \omega \right) = \frac{\omega }{{4\pi i}}\left( {\varepsilon _\infty ^B - 1 + {\sum} {\frac{{\omega _{{\rm{p}},{\rm{n}}}^2}}{{\omega _{{\rm{o}},n}^2 - {\omega ^2} - i{\gamma _n}\omega \mp {\omega _{c,n}}\omega }}} } \right)$$

where *ω*_p,*n*_, *ω*_*o*,*n*,_ *γ*_*n*,_ and *ω*_c,*n*_ are the plasma frequency, energy, linewidth, and cyclotron energy of the *n*-th oscillator, respectively. $\varepsilon _\infty ^B$ represents high-energy contributions to the dielectric constant from energy range higher than all the oscillators. In our analysis, we choose *ω*_o,*n*_ = 0 for all oscillators. The magneto-Drude-Lorentz model ensures that the real and imaginary parts of optical conductivity are constrained by KK relations, therefore it is commonly used to describe LL transitions including CR. The *xx* and *xy* components of the optical conductivity are: ${\sigma _{xx}}\left( \omega \right) = \left[ {{\sigma _ + }\left( \omega \right) + {\sigma _ - }\left( \omega \right)} \right]/2$, ${\sigma _{xy}}\left( \omega \right) = \left[ {{\sigma _ + }\left( \omega \right) - {\sigma _ - }\left( \omega \right)} \right]/2i$. The dielectric function is ${\varepsilon _ \pm }\left( \omega \right) = 1 + \frac{{4\pi i{\sigma _ \pm }\left( \omega \right)}}{\omega }$. The complex reflectivity is given by ${r_ \pm }\left( \omega \right) = \frac{{1 - \sqrt {{\varepsilon _ \pm }\left( \omega \right)} }}{{1 + \sqrt {{\varepsilon _ \pm }\left( \omega \right)} }}$. Finally, the measured reflectance spectrum is given by:

4 $$R\left( \omega \right) = \frac{{{{\left| {{r_ + }\left( \omega \right)} \right|}^2} + {{\left| {{r_ - }\left( \omega \right)} \right|}^2}}}{2}$$

The experimental *R*(*ω*, *B*) data were fit with model reflectance spectrum calculated from Eq. (4). In principle, a complete determination of both *σ*_+_(*ω*) and *σ*_−_(*ω*) cannot be achieved without Kerr rotation measurements. However, after trying numerous versions of *σ*_+_(*ω*) and *σ*_−_(*ω*) to fit *R*(*ω*, *B*), we find that good fits always yield quite similar Re *σ*_*xx*_(*ω*) spectra in the energy range of 75–350 meV. Qualitatively, because of the small changes in *R*(*ω*, *B*)*/R*(*ω*, *B* = 0 T) (less than 5%) and the relatively flat spectral feature in *R*(*ω*, *B* = 0 T) in 75–350 meV, the resonances in *R*(*ω*, *B*) are mathematically related to peaks in Re *σ*_*xx*_(*ω*) at very similar energies, which allows us to determine Re *σ*_*xx*_(*ω*) with good accuracy in this energy range. On the other hand, due to the strong energy dependence of the spectral features in *R*(*ω*, *B* = 0 T) below 50 meV and much larger changes in *R*(*ω*, *B*)*/*R(*ω*, *B* = 0 T) (~20%), it is impossible to accurately determine Re *σ*_*xx*_(*ω*) in the low-energy range. Therefore, we use one oscillator at $\omega _{\rm{c}}^{{\rm{ss}}}$ based on theoretical results on the CR of the SS in TCIs^32^ and several much weaker oscillators to simulate the *R*(*ω*, *B*) spectra below 60 meV, employing the magneto-Drude-Lorentz model. While our discussions are mainly focused on the Re *σ*_*xx*_(*ω*) spectrum, the real and imaginary parts of *σ*_*xx*_(*ω*), *σ*_*xy*_(*ω*), *σ*_*+*_(*ω*), and *σ*_*−*_(*ω*) in representative models are displayed and discussed in Supplementary Note 2.

Our model is a good approximation for investigating Pb_1−*x*_Sn_*x*_Se (*x* = 0.23–0.25), which has a large SS penetration depth. The length scale of the penetration depth (spatial spread) of the SS into the bulk can be estimated as ${\lambda _{{\rm{SS}}}}\sim\hbar v_{\rm{F}}^{{\rm{SS}}}/{\it{\Delta }}$, where $v_{\rm{F}}^{{\rm{SS}}}$ is the Fermi velocity of the Dirac fermions of the SS and *Δ* is the bulk band gap^12^. *λ*_SS_ can be large for small values of *Δ* and even goes to infinity with *Δ* = 0. The value of *λ*_SS_ in Pb_1−*x*_Sn_*x*_Se can be estimated from a comparison with topological insulator Bi_2_Se_3_, whose *λ*_SS_ has been found to be ~2.5 nm (2.5 quintuple layers)^46^. The bulk band gap for our sample is about 1/5 of that of Bi_2_Se_3_ and the two materials have quite similar $v_{\rm{F}}^{{\rm{SS}}}$ values^46^, so the length scale of *λ*_SS_ is expected to be ~12.5 nm. Moreover, theoretical calculations^12^ have shown that the percentage of the SS wavefunction in the topmost layer for Pb_1−*x*_Sn_*x*_Se (*x* = 0.23–0.25) is ~0.5%, which suggests that *λ*_SS_ is much larger than 200 layers or 60 nm based on the lattice constant ~0.3 nm. On the other hand, the far-IR range of 7–25 meV is the main focus of our discussions on the SS, where the averaged length scale of the IR penetration depth estimated from our data is *δ*~13 ± 4 nm, which is comparable to (or smaller than) the length scale of *λ*_SS_. Our analysis employs a model with two main approximations: first, both the SS and bulk states exist within the entire IR penetration depth without a sharp surface/bulk interface, which is justified since *δ*~*λ*_SS_ in Pb_1−*x*_Sn_*x*_Se in the frequency range of interest; second, as an approximation for studying spatially averaged properties of the SS, the model assumes a uniform *n*_SS_ for the SS. With these approximations the IR data can be analyzed using the methods detailed above. Regarding SS penetration depth, Pb_1−*x*_Sn_*x*_Se (*x* = 0.23–0.25) is very different from Z_2_ topological insulators; in the latter materials *λ*_SS_ << *δ* and the IR data can be analyzed using a multilayer model with distinct surface and bulk layers^45, 47^.

### Experimental uncertainty analysis

The uncertainties of the band gap *Δ* and Fermi velocity *v*_F_ of the bulk states are estimated based on the variations of their values from fitting different LL transitions and a confidence interval of 95% in the least squares fit. The uncertainty of *E*_F_ of the bulk states is estimated from the uncertainties in defining *E*_inter_ in the *σ*_1_(*ω*) spectrum or the magnetic field below which the transition LL_−0_→LL_+1_ disappears. The uncertainty of the total Drude SW, SW_total_, is discussed in Supplementary Note 5. In the analysis for data in magnetic field, the uncertainties of the SW, SW_SS_, and scattering rate 1/*τ*_SS_ of the CR mode of the SS represent the range of these parameters that can be used to reproduce the *R*(*ω*, *B*) data. The uncertainties of all other quantities discussed in the main text are calculated using standard formulas for propagation of uncertainty.

### Data availability

The data that support the findings of this study are available from the corresponding author upon reasonable request.

## Electronic supplementary material

Supplementary Information
