# Erratum: Observation of ultrahigh mobility surface states in a topological crystalline insulator by infrared spectroscopy

**DOI:** 10.1038/s41467-017-01354-1

**Published:** 2017-10-12

**Authors:** Ying Wang, Guoyu Luo, Junwei Liu, R. Sankar, Nan-Lin Wang, Fangcheng Chou, Liang Fu, Zhiqiang Li

**Affiliations:** 1 National High Magnetic Field Laboratory, Tallahassee, FL 32310 USA; 2 College of Physical Science and Technology, Sichuan University, Chengdu, Sichuan 610064 China; 3 Department of Physics, Massachusetts Institute of Technology, Cambridge, MA 02139 USA; 4 Department of Physics, Hong Kong University of Science and Technology, Clear Water Bay, Hong Kong, China; 5 Center for Condensed Matter Sciences, National Taiwan University, Taipei, 10617 Taiwan; 6 Institute of Physics, Academia Sinica, Taipei, 11529 Taiwan; 7 International Center for Quantum Materials, School of Physics, Peking University, Beijing, 100871 China

*Nature Communications* **8**:366 10.1038/s41467-017-00446-2; Article published online: 28 August 2017

This Article contains two typesetting errors. In the penultimate sentence of the first paragraph of the Results section, the equation should be ‘$\alpha _0(\omega ) \propto \sqrt {\hbar \omega - \Delta } /\hbar \omega$’, not ‘$\alpha _0(\omega ) \propto \sqrt {\hbar \omega - \Delta /\hbar \omega }$’. In Fig. 4a, the variable marking the top dashed line should be ‘$E_{{\mathrm{H}}1}^{{\mathrm{DP}}}$’, not ‘$E_{{\mathrm{H}}2}^{{\mathrm{DP}}}$’.

