# Supplementary material for: Observation of ultrahigh mobility surface states in a topological crystalline insulator by infrared spectroscopy
Source: Nat Commun. 2017 Aug 28;8:366. doi: 10.1038/s41467-017-00446-2 (PMC5573725; doi:10.1038/s41467-017-00446-2)
Supplement: Supplementary file 1 — Supplementary Information [file 41467_2017_446_MOESM1_ESM.pdf]

File name: Supplementary Information
Description: Supplementary Figures, Supplementary Tables, Supplementary Notes and Supplementary References

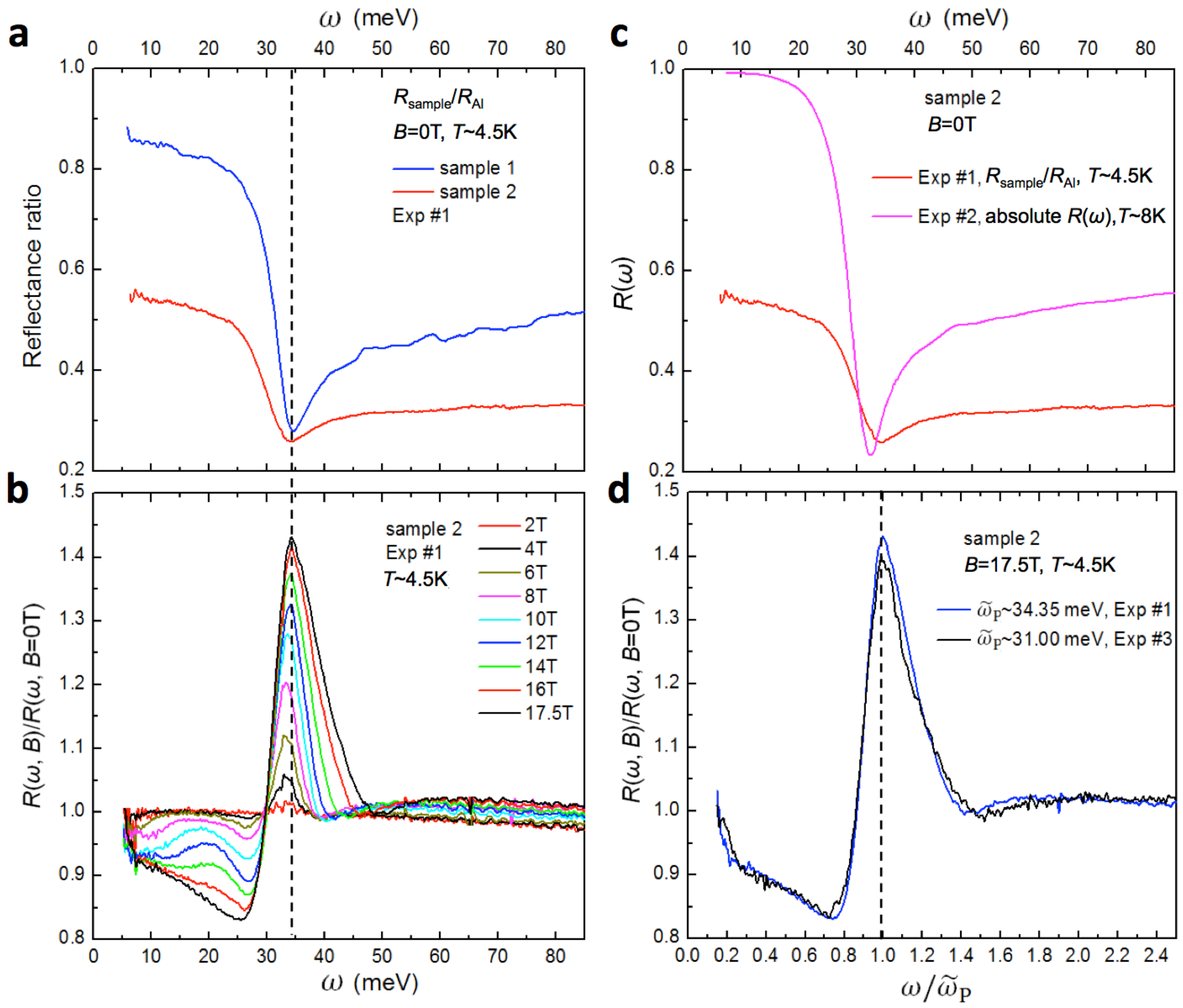

Supplementary Figure 1 | Reflectance spectra of $Pb_{1-x}Sn_xSe$ at zero and high magnetic fields. **a**, The reflectance ratio spectra $R_{\mathrm{sample}}/R_{\mathrm{Al}}$ for two $Pb_{1-x}Sn_xSe$ samples at zero field obtained in Exp #1. **b**, The magneto-reflectance ratio spectra $R(\omega, B)/R(\omega, B = 0\mathrm{T})$ for sample 2 obtained in Exp #1 (sample 1 shows similar results). The dashed line in **a** and **b** shows the screened plasma frequency $\tilde{\omega}_{\mathrm{P}} = \omega_{\mathrm{P}}/\sqrt{\varepsilon_\infty}$. **c**, The reflectance ratio spectrum $R_{\mathrm{sample}}/R_{\mathrm{Al}}$ obtained in Exp #1 and absolute $R(\omega)$ spectrum obtained in Exp #2 for sample 2 in zero field. **d**, The magneto-reflectance ratio spectra $R(\omega, B)/R(\omega, B = 0\mathrm{T})$ at a representative field (B=17.5T) for sample 2 in Exp #1 and #3 displayed in a $\omega/\tilde{\omega}_{\mathrm{P}}$ plot.

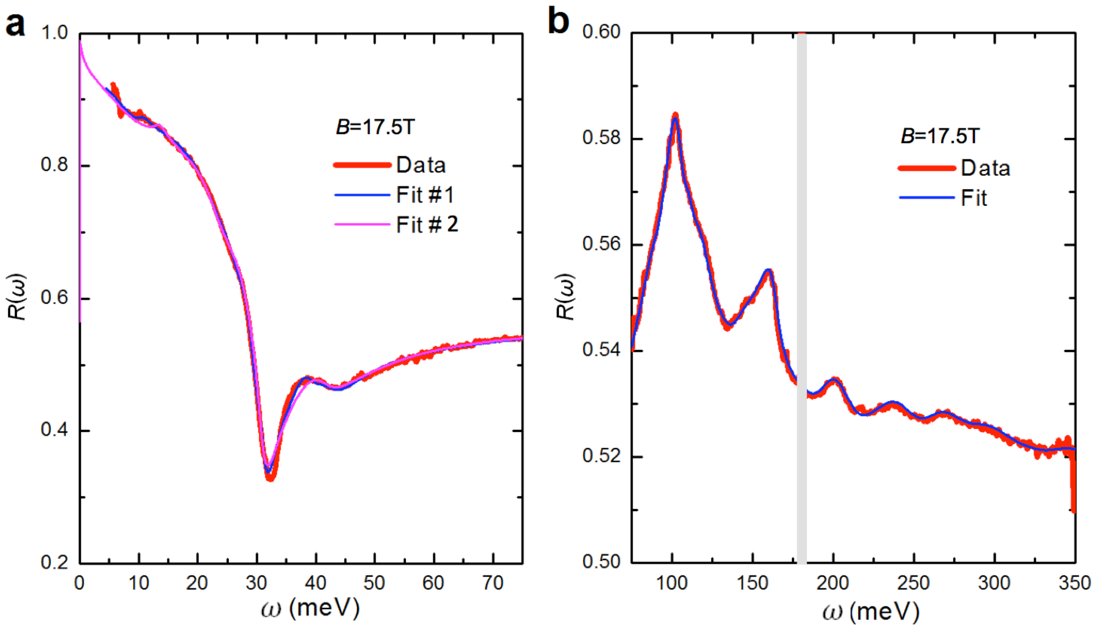

Supplementary Figure 2 | Experimental and model reflectance spectra at *B*=17.5 T. The spectra Fit #1 and #2 in panel **a** are obtained using model #1 and #2 shown in Supplementary Figures 3 and 4, respectively. Model #1 is the model presented in the main text. The fit spectra in panel **b** are obtained using model #1. The vertical axis is shown in different scales in **a** and **b** to highlight small features in $R(\omega, B)$. The gray area around 175 meV is the energy range in which no data can be obtained due to the IR absorption of the optical window in our setup.

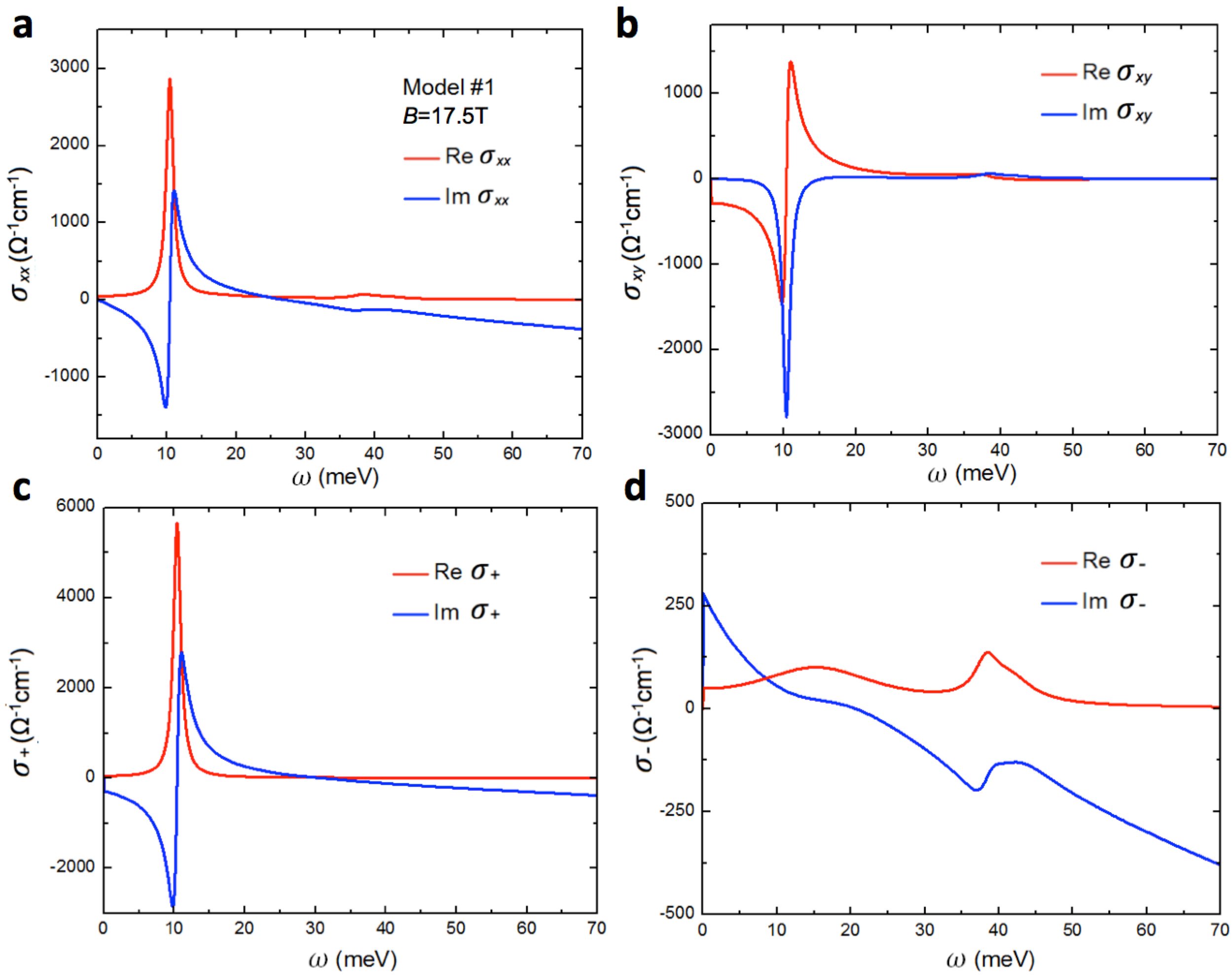

Supplementary Figure 3 | Real and imaginary parts of optical conductivity from model #1. All parameters for model #1 are summarized in Supplementary Table 1, which is the model presented in the main text.

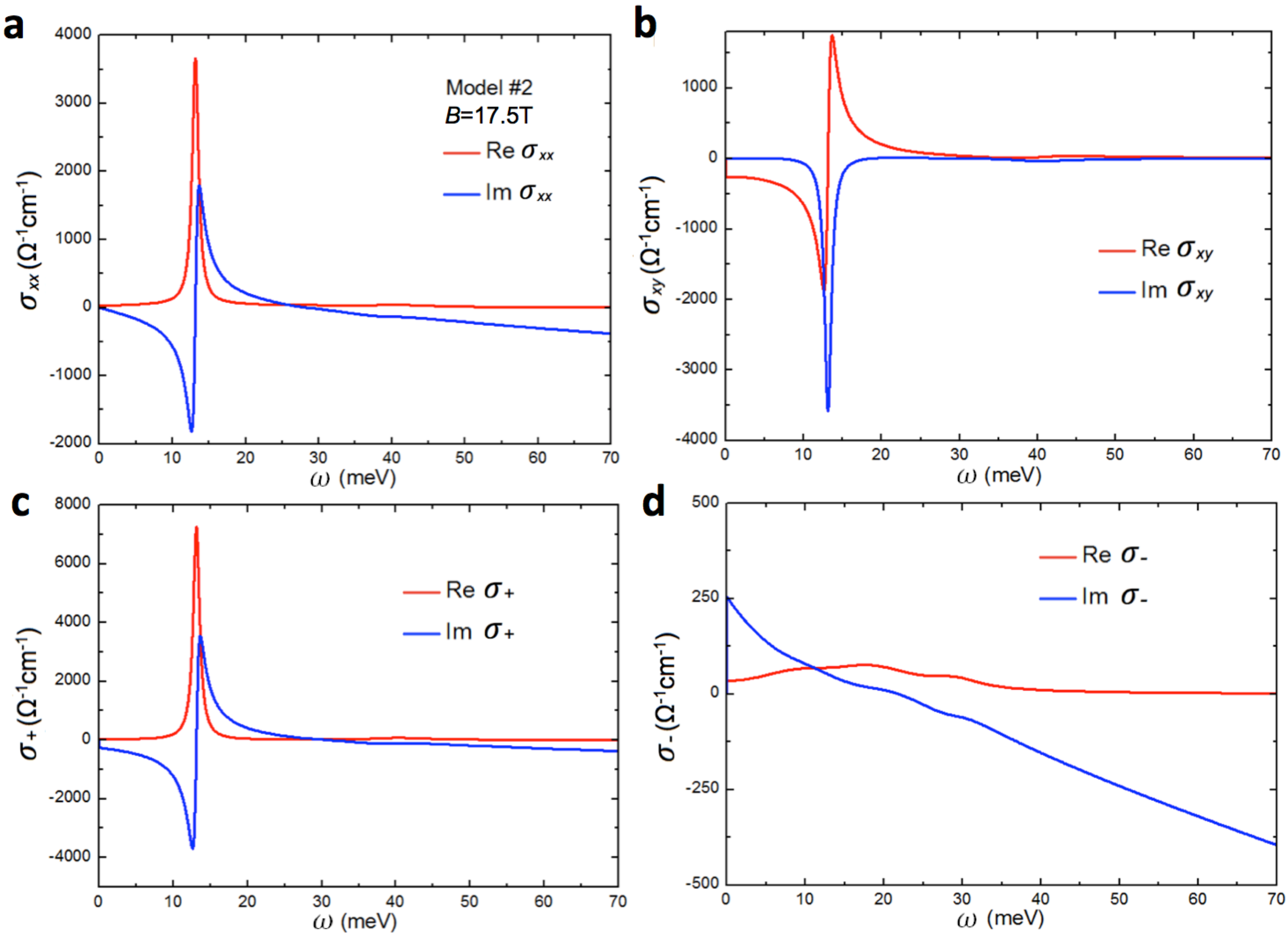

Supplementary Figure 4 | Real and imaginary parts of optical conductivity from model #2. All parameters for model #2 are summarized in Supplementary Table 2.

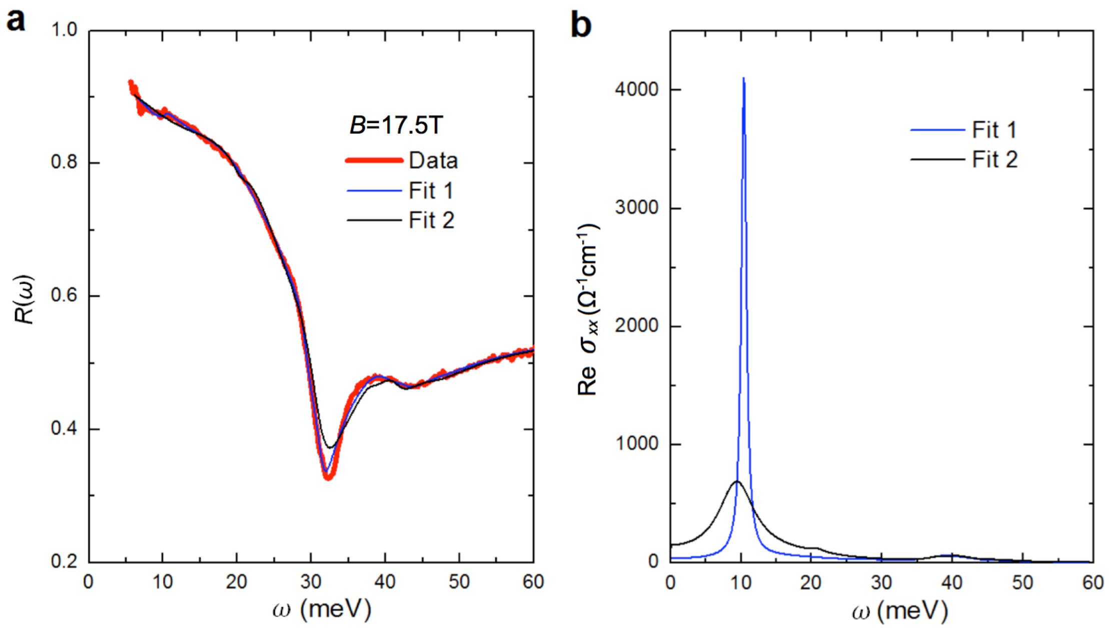

Supplementary Figure 5 | Experimental and model reflectance spectra at *B*=17.5 T. a, Experimental $R(\omega, B)$ spectra and two model spectra. **b**, The Re $\sigma_{xx}(\omega, B)$ spectra in two models used to generate the model spectra in **a**. Blue curves: $1/\tau_{SS}$=0.6 meV. Black curves: $1/\tau_{SS}$=3 meV.

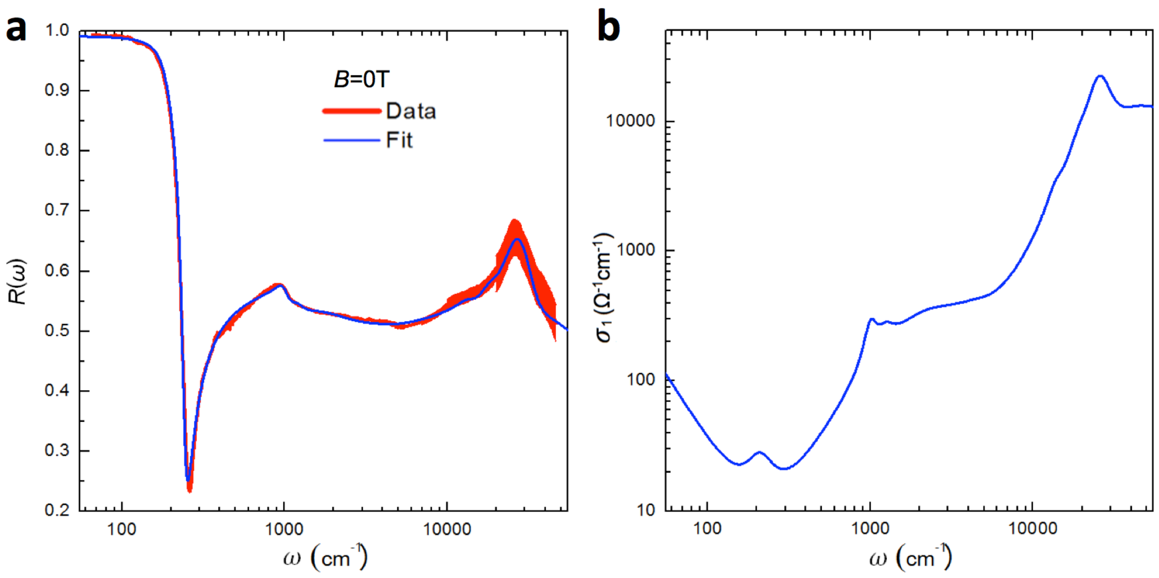

Supplementary Figure 6 | Drude-Lorentz fit of reflectance spectrum at zero field. a, Experimental $R(\omega)$ spectrum in zero field and a typical fit using the Drude-Lorentz model. The thickness of the experimental spectrum represents the estimated uncertainty. **b**, The corresponding $\sigma_1(\omega)$ spectrum. The parameters for all oscillators in the model are summarized in Supplementary Table 3.

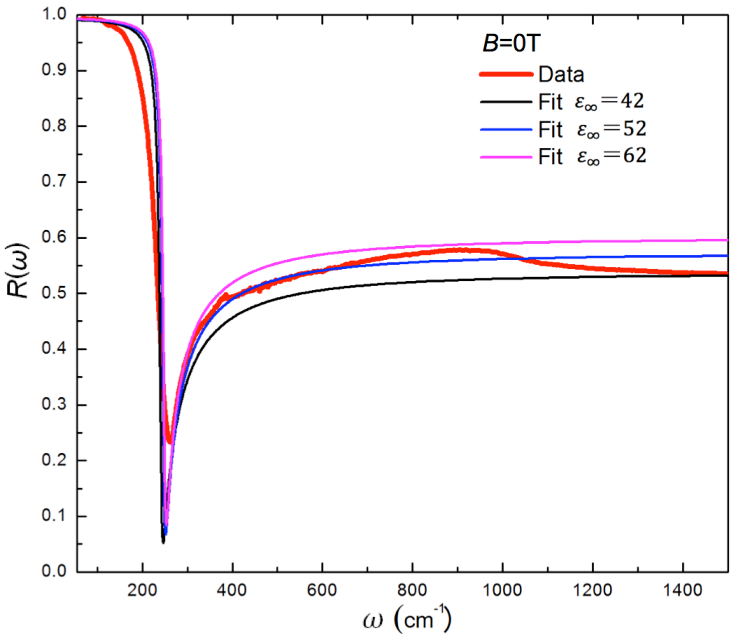

Supplementary Figure 7 | Experimental and model reflectance spectra at zero field, The model $R(\omega)$ spectra are calculated from the Drude model $\sigma(\omega) = \frac{\omega}{4\pi i}(\varepsilon_\infty - 1 - \frac{\omega_\mathrm{P}^2}{\omega^2 + i\gamma\omega})$, where ε_∞ represents all electronic contributions to the dielectric constant other than the Drude conductivity. The model spectra with different values of ε_∞ are generated using $\gamma = 7$ cm^{-1} and $\omega_\mathrm{P} = \widetilde{\omega}_\mathrm{P}\sqrt{\varepsilon_\infty}$ with $\widetilde{\omega}_\mathrm{P} \sim 260$ cm^{-1}. The $R(\omega)$ spectrum above $\widetilde{\omega}_\mathrm{P}$ increases with increasing value of ε_∞.

Supplementary Table 1 | Parameters for model #1 at *B*=17.5T. The model spectra are presented in the main text and Supplementary Figure 3. These parameters are used in Eq. (3) of the main text. $\varepsilon_\infty^B = 49.24$, and $\omega_{\mathrm{o},n} = 0$ for all oscillators.

**Energy** $\omega_{\mathrm{c},n}$ **(cm^{-1})**	**Plasma frequency** $\omega_{\mathrm{p},n}$ **(cm^{-1})**	**Linewidth** γ_n **(cm^{-1})**
124	689	82
309	276	15
334	325	30
-84	1301	5
-218	1117	19
-244	81	12

Supplementary Table 2 | Parameters for model #2 at *B*=17.5T. The model spectra are presented in Supplementary Figure 4. These parameters are used in Eq. (3) of the main text for magneto-Drude-Lorentz model #2, which is another representative model. $\varepsilon_{\infty}^{B} = 49.73$, and $\omega_{\mathrm{o},n} = 0$ for all oscillators.

**Energy** $\omega_{\mathrm{c},n}$ **(cm^{-1})**	**Plasma frequency** $\omega_{\mathrm{p},n}$ **(cm^{-1})**	**Linewidth** γ_n **(cm^{-1})**
70	338	50
148	459	59
235	200	30
-106	1351	4.2
-230	151	25
-325	350	30
-370	226	55

Supplementary Table 3 | Parameters for Drude-Lorentz model at zero field. The model spectra are presented in Supplementary Figure 6. These parameters are used in Eq. (3) of the main text with $\omega_{c,n} = 0$ for all oscillators, which is the zero-field Drude-Lorentz model. $\varepsilon_\infty^B = 2.733$.

**Energy** $\boldsymbol{\omega_{0,n}}$ **(cm^{-1})**	**Plasma frequency** $\boldsymbol{\omega_{p,n}}$ **(cm^{-1})**	**Linewidth** $\boldsymbol{\gamma_n}$ **(cm^{-1})**
0	1730.98	7
209.78	276.55	93.41
1005.90	1325.32	201.24
1241.20	1326.11	382.13
2313.25	7785.34	3572.83
4310.72	4058.41	3426.14
13800	14239.48	4050
18960.71	24857.39	5535.32
25645.54	104447.11	10000.87
44100.83	139379.84	39529.24
71803.38	164841.51	57286.62

Supplementary Note 1. *R(ω, B)* spectra of $Pb_{1-x}Sn_xSe$ and discussion on Se vacancy defects

The magneto-reflectance ratio $R(\omega,B)/R(\omega,B=0\text{T})$ and the zero-field reflectance $R(\omega)$ for two $Pb_{1-x}Sn_xSe$ (x=0.23-0.25) samples were measured in 3 different experiments (Exp #1-3 in sequence) with several months between one another. All the data in the main text are from sample 2. Supplementary Figure 1a shows the reflectance ratio data $R_{\text{sample}}/R_{\text{Al}}$ at zero field obtained in Exp #1, which is the reflectance of sample divided by that of a reference aluminum mirror. Because of the lack of in situ gold coating in this experiment, the $R_{\text{sample}}/R_{\text{Al}}$ spectra are not absolute $R(\omega)$. Nevertheless, the plasma minimum (dip in $R_{\text{sample}}/R_{\text{Al}}$) at the screened plasma frequency $\tilde{\omega}_{\text{P}} = \omega_{\text{P}}/\sqrt{\varepsilon_\infty}$ shown in Supplementary Figure 1a should be fairly accurate owing to the frequency independent $R(\omega)$ of aluminum in this range. Here, the bare plasma frequency ω_{P} is given by $\omega_{\text{P}}^2 = 4\pi e^2 n/m$, ε_∞ represents all high-energy contributions to the dielectric constant other than the free carrier contribution. The data in Supplementary Figures 1a and 1b show that the peak in the $R(\omega,B)/R(\omega,B=0\text{T})$ spectrum is exactly at the plasma minimum frequency $\tilde{\omega}_{\text{P}}$ at zero field measured in the same experiment.

In order to obtain the absolute $R(\omega)$ spectrum at zero field, we performed Exp #2 with in situ gold coating technique, after which $R(\omega,B)/R(\omega,B=0\text{T})$ were measured again in Exp #3. We find that $\tilde{\omega}_{\text{P}}$ changes slightly from ~34.35 meV to ~32.49 meV (Supplementary Figure 1c), and then to ~31 meV in Exp #1-3. This observation probably arises from self-doping due to Se vacancies caused by the so-called "Se loss phenomenon" in selenides, namely, thermal energy assisted surface Se atom escape, which is very common in selenides such as $TiSe_2$, SnSe and Bi_2Se_3 as discussed in ref. [1] and references therein. The sample is cooled down to ~4.5K or ~10K then warmed up to 300K for multiple times during different measurements, between which it is briefly exposed to air. Every thermal cycling could introduce Se loss, especially for the Se atoms at the surface with the weakest

chemical bonding. The Se loss effect could lead to changes in the carrier density n and therefore the change of $\tilde{\omega}_P$ with time, since $\tilde{\omega}_P \propto \sqrt{n}$. Importantly, we find that the frequencies of the features in $R(\omega, B)$ / $R(\omega, B$ =0T) below 60 meV change proportionally with $\tilde{\omega}_P$. As shown in Supplementary Figure 1d, $R(\omega, B)/R(\omega, B=$ 0T) measured in Exp #1 and #3 remain the same within experimental uncertainty (1%-1.5%) if they are plotted in a $\omega/\tilde{\omega}_P$ plot. The scaling results in Supplementary Figure 1d demonstrate the robustness of the observed features in $R(\omega, B)/R(\omega, B =$ 0T) despite the small change of $\tilde{\omega}_P$ with time, which is an extrinsic effect. Also, the observed main features in $R(\omega, B)/R(\omega, B=$ 0T) above 60 meV change little from Exp #1 to Exp #3, which we believe is due to the fact that these features arise from interband Landau level transitions and are therefore relatively insensitive to the change of carrier density.

There are two methods to account for the small change of $\tilde{\omega}_P$ with time in our data. First, one can use the absolute $R(\omega, B=$ 0T) spectrum obtained in Exp #2 (Supplementary Figure 1c), and use the $R(\omega, B)/R(\omega, B=$ 0T) spectra in Exp #1 (Supplementary Figure 1b) after scaling the frequency by the ratio of the $\tilde{\omega}_P$ in Exp #2 and #1 below 60 meV. The validity of this approach is justified by the scaling results shown in Supplementary Figure 1d, especially because Exp #2 is performed between Exp #1 and #3. As the second approach, the $R(\omega, B)/R(\omega, B=$ 0T) in Exp #1 will be used and the corresponding $R(\omega, B=$ 0T) spectrum can be obtained from that in Exp #2 (Supplementary Figure 1c) after scaling the frequency by the ratio of the $\tilde{\omega}_P$ in Exp #1 and #2 below 60 meV. This is justified because the $R(\omega, B=$ 0T) spectrum obtained in Exp #2 can be satisfactorily described by the Drude model, and the $R(\omega)$ spectra in the Drude model with different $\tilde{\omega}_P$ will be identical if they are all displayed a $\omega/\tilde{\omega}_P$ plot. In both methods, the $R(\omega, B)$ spectra are obtained by multiplying the $R(\omega, B)/R(\omega, B=$ 0T) spectra by $R(\omega, B=$ 0T). In the main text, data obtained using the first method is presented and discussed. The data obtained from the second method yield the same conclusions discussed in the main text.

It has been a longstanding challenge in the research of selenides to control of the sample quality. Even for samples with the same nominal Pb/Sn ratio, the actual carrier density is sample dependent, which depends strongly on the self-doping effect of Se vacancy defects in each crystal. In the Bridgman crystal growth, the details can be slightly different for every batch, such as vacuum control and the specific solidification temperature at the solid-liquid interface. Moreover, the vacancy defect has higher density closer to the surface. All of these factors fluctuate in each growth, which leads to sample-dependent defect density and therefore carrier density. The samples used in our IR study are from a batch with low carrier density, but the carrier density is batch dependent even for the same nominal Pb/Sn ratio.

Supplementary Note 2. Details on $\sigma_{xx}(\omega)$ and $\sigma_{xy}(\omega)$ in our analysis of $R(\omega, B)$ and CR mode of the surface states

As discussed in the main text, the reflectance data $R(\omega, B)$ were analyzed using the magneto-Drude-Lorentz model. This model ensures that the real and imaginary parts of optical conductivity are constrained by Kramers–Kronig relations, therefore it is commonly used to describe Landau level transitions including cyclotron resonance (CR). This model is among the most used methods of parametrization of optical response functions including optical conductivity. The physical meaning of each individual oscillator in the model may not be always clear, but the meaningful result is the overall optical conductivity obtained from all oscillators, as discussed in details in reference 28 of the main text and references therein. In our analysis, we use one oscillator at $\omega_c^{ss} = eB/m_{ss}$ based on theoretical study of Landau levels of the SS in TCIs (reference 32 of the main text) and several much weaker oscillators to simulate the $R(\omega, B)$ spectra below 60 meV, which represent a parametrization of the overall optical conductivity as explained above.

Supplementary Figure 2 displays the experimental $R(\omega, B)$ spectra together with two representative model spectra at B=17.5 T. The real and imaginary parts of $\sigma_{xx}(\omega)$, $\sigma_{xy}(\omega)$, $\sigma_{+}(\omega)$ and $\sigma_{-}(\omega)$ in representative models are shown in Supplementary Figure 3 and 4, where model #1 is corresponding to the model for B=17.5T in Fig 4c of the manuscript. The parameters for all oscillators in each model are summarized in Supplementary Tables 1 and 2.

Depending on the width and spectral weight of the oscillators used in our analysis and their distributions in $\sigma_{+}(\omega)$ and $\sigma_{-}(\omega)$, we find that a range of $\omega_{\mathrm{c}}^{\mathrm{ss}}$ values with different possible $\sigma_{xx}(\omega)$ spectra can reproduce the spectral feature in $R(\omega, B)$ below 25 meV and its evolution with B field. Two representative models are shown in Supplementary Figure 3 and 4. Specifically, simulated $\sigma_{xx}(\omega)$ spectra using m_{ss} values in the range of 0.15-0.19 m_e for the CR mode yield model $R(\omega, B)$ spectra that can fit the experimental data with the same quality as those shown in Supplementary Figure 2a within experimental uncertainties. As discussed in the main text, scanning tunneling microscopy (STM) and angle-resolved photoemission spectroscopy (ARPES) experiments [2-4] suggest that the effective mass for the SS associated with the Dirac cone at $E_{\mathrm{H2}}^{\mathrm{DP}}$ is $m_{\mathrm{ss}} = E_{\mathrm{F}}^{\mathrm{SS}}/(\bar{v}_{\mathrm{F}}^{\mathrm{SS}})^2$ =0.15±0.015 m_e. Therefore the effective mass values m_{ss} estimated from our IR experiments are consistent with those inferred from STM and ARPES measurements within 15% (Fig. 4d of the main text). This excellent agreement strongly supports our identification of the CR mode of SS discussed in the main text. The small deviation between our results and STM and ARPES measurements may arise from the difference in Fermi energy in different samples. Moreover, the spectral feature in $R(\omega, B)$ data below 25 meV are well below the energy range of all allowed LL transitions from the bulk states, so it can only be assigned to the SS.

The lineshape of $R(\omega, B)$ is determined by real and imaginary parts of $\sigma_{xx}(\omega)$ and $\sigma_{xy}(\omega)$ based on Eq.(4) in the main text. Although there is a sharp peak due to SS in Re $\sigma_{xx}(\omega)$, the

contributions from all real and imaginary parts of $\sigma_{xx}(\omega)$ and $\sigma_{xy}(\omega)$ in Supplementary Figure 3 and 4 lead to a relatively smooth lineshape for $R(\omega, B)$ in this spectral range, which is a consequence of the spectral properties (lineshapes) of optical response functions typically described by magneto-Drude-Lorentz oscillators.

Supplementary Note 3. Comparing $R(\omega, B)$ data with the conventional magnetoplasma effect in semiconductors

Conventional doped semiconductors exhibit the so-called magnetoplasma effect in magnetic field [5, 6]: the plasma minimum (edge) in the $R(\omega)$ spectra in zero field splits into two minimums (edges) in $R(\omega, B)$ in magnetic field, with the high (low) energy edge moving to higher (lower) energies with increasing field. This effect is observed in the regime of $\omega_c \ll \tilde{\omega}_P$, where $\tilde{\omega}_P$ is the frequency of the plasma minimum in the $R(\omega)$. Such a behavior in $R(\omega, B)$ spectrum arises from a single CR mode at ω_c in the optical conductivity $\sigma_{xx}(\omega, B)$ that is well separated in energy from other resonances such as interband LL transitions [5, 6]. In our measurements of $Pb_{1-x}Sn_xSe$, the observed features in $R(\omega, B)$ below 50 meV are entirely different from the magnetoplasma effect described above. Our observation results from the overall contributions of two resonances in this energy range: the CR mode of the SS and the $LL_{+0} \rightarrow LL_{+1}$ transition from the bulk states (Fig. 4c of the main text). We stress that the $LL_{+0} \rightarrow LL_{+1}$ transition around 30-40 meV is the LL transition with the lowest energy from the bulk states, which can not produce the dramatic changes below ~25 meV in $R(\omega, B)$ with increasing magnetic field. The latter observation suggests the existence of a low energy resonance below the $LL_{+0} \rightarrow LL_{+1}$ transition of the bulk, which we demonstrate to be the CR mode of the SS.

Supplementary Note 4. Scattering rate and mobility of the surface states

As illustrated in Supplementary Figure 5, the width of the dip feature around 32 meV in $R(\omega, B)$ is directly related to the width of the CR mode of the SS in $\mathrm{Re}\,\sigma_{xx}(\omega)$ spectra. Therefore, the very narrow dip feature around 32 meV in the $R(\omega, B)$ data suggests that the scattering rate for the SS $1/\tau_{\mathrm{SS}}$ is very low. We find that $\mathrm{Re}\,\sigma_{xx}(\omega)$ spectra with $1/\tau_{\mathrm{SS}}\sim 1.2 \pm 0.6$ meV for the surface CR mode can reproduce the $R(\omega, B)$ data. As shown in Supplementary Figure 5, larger values of $1/\tau_{\mathrm{SS}}$ are inconsistent with the narrow dip feature in $R(\omega, B)$. This estimation of $1/\tau_{\mathrm{SS}}$ allows us to estimate the mobility of the SS as discussed in the main text.

It is instructive to compare the surface mobility in TCIs to the mobility of graphene [7-9] since both materials feature massless Dirac fermions. In a simple Drude model, the mobility of carriers in graphene is given by [8]:

$$\mu = e v_{\mathrm{F}} \tau / (\hbar k_{\mathrm{F}}) = e\tau/m, \qquad (1)$$

where $m = E_{\mathrm{F}}/v_{\mathrm{F}}^2$. In a previous study [9] of graphene samples with carrier density $n\sim 4.7\times10^{12}$ cm^{-2} (corresponding to $m\sim 0.04\ m_e$), the scattering rate is found to be $1/\tau\sim 30$ meV based on IR study of Landau level transitions, which yields an IR mobility of $\mu_{\mathrm{IR}}\sim 5{,}700$ $\mathrm{cm}^2\,\mathrm{V}^{-1}\,\mathrm{s}^{-1}$ based on Supplementary Eq. (1). The estimated μ_{IR} is in reasonable agreement with the reported DC mobility $\mu_{\mathrm{DC}}\sim 4{,}000$ $\mathrm{cm}^2\,\mathrm{V}^{-1}\,\mathrm{s}^{-1}$ [9]. The lower value of μ_{DC} compared to μ_{IR} might arise from disorder effects as discussed in [7]. The good agreement between μ_{DC} and μ_{IR} in graphene [9] supports our estimation of the surface mobility μ_{SS} in TCIs using an equation similar to Supplementary Eq. (1). Moreover, a scattering rate of $1/\tau\sim 2$ meV was observed in IR measurements of graphene/BN with mobility $\mu\sim 50{,}000$ $\mathrm{cm}^2\,\mathrm{V}^{-1}\,\mathrm{s}^{-1}$ [7]. Therefore, the scattering rate and mobility from previous studies of graphene [7,9] provide further support for our estimation of $\mu_{\mathrm{SS}}\sim 40{,}000$ $\mathrm{cm}^2\,\mathrm{V}^{-1}\,\mathrm{s}^{-1}$ in TCIs.

Supplementary Note 5. Analysis of total Drude spectral weight in zero field

We use the Drude-Lorentz model to fit the $R(\omega)$ data in zero field. One typical fit to $R(\omega)$ and the corresponding $\sigma_1(\omega)$ are shown in Supplementary Figure 6 with the parameters for all oscillators in the model summarized in Supplementary Table 3. The optical conductivity $\sigma(\omega)$=$\sigma_1(\omega)$+i$\sigma_2(\omega)$ from Drude-Lorentz fit is consistent with that evaluated from Kramers-Kronig (KK) transformation of $R(\omega)$. The total Drude spectral weight ($\mathrm{SW_{total}}$) can be directly determined by the bare plasma frequency ω_{P} of the Drude oscillator from $\mathrm{SW_{total}} = \frac{\pi\Omega^{-1}}{120}\omega_{\mathrm{P}}^2$, where ω_{P} is in cm^{-1}, Ω is Ohm. The total (observed) plasma frequency ω_{P} for the Drude mode can be obtained from fitting the entire $R(\omega)$ spectrum shown in Supplementary Figure 6a, because it is related to the screened plasma frequency $\widetilde{\omega}_{\mathrm{P}}$ by:

$$\omega_{\mathrm{P}} = \widetilde{\omega}_{\mathrm{P}}\sqrt{\varepsilon_\infty} \qquad (2)$$

where $\widetilde{\omega}_{\mathrm{P}}$~260 cm^{-1} corresponds to the plasma minimum in $R(\omega)$ and ε_∞ is determined by all Lorentzian oscillators obtained from fitting the overall R(ω) spectrum above $\widetilde{\omega}_{\mathrm{P}}$. ε_∞ represents all electronic contributions to the dielectric constant other than the Drude conductivity, which is given by (see for example, [10]):

$$\varepsilon_\infty \equiv \varepsilon_1(\omega)\big|_{\omega\to 0} = 1 + \frac{120}{\pi} P\int_{\omega_0}^{\infty} \frac{\sigma_1(\omega\prime)}{\omega\prime^2-\omega^2}d\omega'\bigg|_{\omega\to 0} \qquad (3)$$

where P denotes the Cauchy principal value, frequencies are in cm^{-1}, σ_1 is in $\Omega^{-1}\mathrm{cm}^{-1}$, and ω_0 is a frequency separating the Drude mode and the interband transitions (ω_0~200 cm^{-1}). The high-energy contribution of $\sigma_1(\omega)$ to ε_∞ is negligibly small because of the denominator of the integrand in Supplementary Eq. (3). Taking into account the uncertainties of $\sigma_1(\omega)$ from both Drude-Lorentz fit and KK transformation of $R(\omega)$ as well as those for ω_0, we estimate ε_∞~45 ± 9 using Supplementary Eq. (3).

In fact, the lineshape and absolute value of the $R(\omega)$ spectrum above $\tilde{\omega}_{\mathrm{P}}$ directly reflects the value of ε_{∞}. Because the $R(\omega)$ spectrum shows a typical metallic behavior near and below $\tilde{\omega}_{\mathrm{P}}$, we can use a simple Drude model to illustrate the dependence of $R(\omega)$ on ε_{∞}. Supplementary Figure 7 displays model $R(\omega)$ spectra calculated from the Drude model $\sigma(\omega) = \frac{\omega}{4\pi i}(\varepsilon_{\infty} - 1 - \frac{\omega_{\mathrm{P}}^2}{\omega^2 + i\gamma\omega})$ with different values of ε_{∞}, which shows that the $R(\omega)$ spectrum above $\tilde{\omega}_{\mathrm{P}}$ increases with increasing value of ε_{∞}. Although $R(\omega)$ spectra from the Drude model in Supplementary Figure 7 only intends to qualitatively illustrate the effect of ε_{∞}, it shows that the absolute value of the experimental $R(\omega)$ spectrum above $\tilde{\omega}_{\mathrm{P}}$ can be reproduced by $\varepsilon_{\infty}{\sim}52 \pm 10$, which is in good agreement (within 15%) with the result from our full analysis ($\varepsilon_{\infty}{\sim}45 \pm 9$) using Drude-Lorentz model. Therefore, our analysis demonstrates that $\omega_{\mathrm{P}} = \tilde{\omega}_{\mathrm{P}}\sqrt{\varepsilon_{\infty}}{\sim}(1744 \pm 174)$ cm^{-1} and $\mathrm{SW_{total}} \approx (7.9 \pm 1.6)\times 10^4\ \Omega^{-1}\mathrm{cm}^{-2}$. This method of evaluating $\mathrm{SW_{total}}$ doesn't rely on the integral $\int_0^{\omega_0}\sigma_1(\omega)d\omega$, so it is not limited by the lack of information on $\sigma_1(\omega)$ for the Drude mode below ~7meV.

Supplementary References

1. Huang, S. H., Shu, G. J., Pai, W.W., Liu, H. L.& Chou, F. C. Tunable Se vacancy defects and the unconventional charge density wave in 1T−$TiSe_{2-\delta}$. *Phys. Rev. B* **95**, 045310 (2017).
2. Serbyn, M. & Fu, L. Symmetry breaking and Landau quantization in topological crystalline insulators. *Phys. Rev. B* **90,** 035402 (2014).
3. Okada, Y. *et al*. Observation of Dirac node formation and mass acquisition in a topological crystalline insulator. *Science* **341**, 1496–1499 (2013).
4. Neupane, M. *et al*. Topological phase diagram and saddle point singularity in a tunable topological crystalline insulator. *Phys. Rev. B* **92,** 075131 (2015).
5. Lax, B. & Wright, G.B. Magnetoplasma Reflection in Solids. *Phys. Rev. Lett.* **4**, 16 (1960).

6. Palik, E. D. & Furdyna, J. K. Infrared and microwave magnetoplasma effects in semiconductors. *Rep. Prog. Phys.* **33**, 1193-1322 (1970).

7. Chen, Z. G. *et al*. Observation of an intrinsic bandgap and Landau level renormalization in graphene/boron-nitride heterostructures. *Nat. Commun.* **5,** 4461 (2014).

8. Basov, D. N., Fogler, M. M., Lanzara, A., Wang, F. & Zhang, Y. Colloquium: Graphene spectroscopy. *Rev. Mod. Phys.* **86**, 959-994 (2014).

9. Jiang, Z. *et al*. Infrared spectroscopy of Landau levels of graphene. *Phys. Rev. Lett.* **98**, 197403 (2007).

10. Hwang, J.,Timusk T. & Gu, G. D. Doping dependent optical properties of $Bi_2Sr_2CaCu_2O_{8+\delta}$. *J. Phys. Condens. Matter* **19**, 125208 (2007).
